# Primary chemotherapy with adriamycin, cisplatin, vincristine and cyclophosphamide in locally advanced thymomas: a single institution experience

**DOI:** 10.1038/sj.bjc.6690773

**Published:** 1999-11

**Authors:** A Berruti, P Borasio, A Gerbino, G Gorzegno, T Moschini, M Tampellini, F Ardissone, M P Brizzi, A Dolcetti, L Dogliotti

**Affiliations:** Dipartimento di Scienze Cliniche e Biologiche, Oncologia Medica, Università di Torino, Torino Italy; Unità Operativa Autonoma di Chirurgia Toracica Unità Operativa Autonoma di Pneumologia, 1–3 Azienda Ospedaliera San Luigi, Regione Gonzole 10, 10043 Orbassano, Italy

**Keywords:** primary chemotherapy, thymome, radiotherapy

## Abstract

From 1990 to 1997, 16 consecutive patients with stage III and IVa invasive thymoma were treated in a single institution with primary chemotherapy consisting in adriamycin (40 mg m^–2^), cisplatin (50 mg m^–2^) administered intravenously on day 1, vincristine (0.6 mg m^–2^) on day 2 and cyclophosphamide (700 mg m^–2^) on day 4 (ADOC). The courses were repeated every 3 weeks. The aim was to evaluate the impact of this cytotoxic regimen with respect to response rate, per cent of patients radically resected, time to progression and overall survival. Two complete responses (one clinical and one pathological) and 11 partial responses were observed (overall response rate 81.2%); two patients had stable disease and one progressed. Toxicity was mild as only two patients developed grade III/IV neutropenia and one patient grade III nausea/vomiting. Nine patients were radically resected (five out of ten with stage III, and four out of six with stage IVa). Median time to progression and overall survival was 33.2 and 47.5 months respectively. Three patients were alive and disease free after more than 5 years. The ADOC scheme is highly active and manageable in the treatment of locally advanced thymoma. As a preoperative approach it should be offered to patients not amenable to surgery or to those surgically resectable but with a great deal of morbidity. © 1999 Cancer Research Campaign


*British Journal of Cancer* (1999) **81**(5), 841–845
© 1999 Cancer Research Campaign
Article no. bjoc.1999.0773

### Primary chemotherapy with adriamycin, cisplatin, vincristine and cyclophosphamide in locally advanced thymomas: a single institution experience

**A Berruti[1], P Borasio[2], A Gerbino[1], G Gorzegno[1], T Moschini[3], M Tampellini[1], F Ardissone[2], MP Brizzi[1], A Dolcetti[3] and L Dogliotti[4]**

[1]Dipartimento di Scienze Cliniche e Biologiche, Oncologia Medica, Università di Torino, Torino, Italy; [2]Unità Operativa Autonoma di Chirurgia Toracica, [3]Unità Operativa Autonoma di Pneumologia, [4]1–3 Azienda Ospedaliera San Luigi, Regione Gonzole 10, 10043 Orbassano, Italy

**Summary** From 1990 to 1997, 16 consecutive patients with stage III and IVa invasive thymoma were treated in a single institution with primary chemotherapy consisting in adriamycin (40 mg $m^{-2}$), cisplatin (50 mg $m^{-2}$) administered intravenously on day 1, vincristine (0.6 mg $m^{-2}$) on day 2 and cyclophosphamide (700 mg $m^{-2}$) on day 4 (ADOC). The courses were repeated every 3 weeks. The aim was to evaluate the impact of this cytotoxic regimen with respect to response rate, per cent of patients radically resected, time to progression and overall survival. Two complete responses (one clinical and one pathological) and 11 partial responses were observed (overall response rate 81.2%); two patients had stable disease and one progressed. Toxicity was mild as only two patients developed grade III/IV neutropenia and one patient grade III nausea/vomiting. Nine patients were radically resected (five out of ten with stage III, and four out of six with stage IVa). Median time to progression and overall survival was 33.2 and 47.5 months respectively. Three patients were alive and disease free after more than 5 years. The ADOC scheme is highly active and manageable in the treatment of locally advanced thymoma. As a preoperative approach it should be offered to patients not amenable to surgery or to those surgically resectable but with a great deal of morbidity.

**Keywords:** primary chemotherapy; thymome; radiotherapy

Thymomas are rare neoplasms arising from the epithelial cells in the thymus gland; they represent the most frequent tumours of the anterior mediastinum. Their estimated incidence in Italy is about 0.13/100 000 new cases every year (Verdecchia et al, 1997; Decarli et al, 1998). Thymomas are usually slow growing and have benign-appearing histological findings (Pollak et al, 1992). The malignant behaviour is not based on histological criteria but on macroscopic and microscopic signs of invasiveness. The presence of invasion is considered to be the single most important factor predictive for future behaviour (Verley et al, 1985; Maggi et al, 1986) and forms the basis for the most widely used clinical staging system described by Masaoka (1981). Among patients with invasive thymoma, prognosis has been directly related to clinical Masaoka stage and to the ability to perform a complete excision (Maggi et al, 1991). Conversely, considerable controversies exist regarding the importance of other potential prognostic factors, namely the presence of associated autoimmune disorders, and the histological subtype (Lewys et al, 1987; Pescarmona et al, 1990).

The majority of patients are diagnosed with an encapsulated or non-invasive tumour, while 35–40% have locally advanced or metastatic disease (Maggi et al, 1991). Complete surgical resection is a successful treatment especially in the absence of significant infiltration of the surrounding tissue, yelding to a less than 5% recurrence rate (Maggi et al, 1991; Kohmann, 1997; Schmidt et al, 1997). Surgery is the treatment of choice also in patients with locally advanced disease and should be performed whenever possible (Yagi et al, 1996), even in cases of disease recurrence (Kirschner, 1990; Maggi et al, 1991). Locally advanced thymomas tend to recur after complete resection, therefore adjuvant radiotherapy is often prescribed, although further studies are needed to confirm its benefit (Curran et al, 1988; Urgesi et al, 1992). In large, invasive thymomas, with invasion of major vessels or cardiac structures, complete resection is more difficult to achieve and, despite the improvement of surgery techniques, radical resection is reportedly feasible only in a half of cases bearing stage III and hardly ever in those with stage IV (Maggi et al, 1991).

The role of chemotherapy is controversial and usually confined to patients developing recurrent disease after surgery and radiotherapy (Kohmann, 1997). A few single-institution experiences (Giaccone et al, 1985; Fornasiero et al, 1991; Macchiarini et al, 1991) and two intergroup studies (Loeher et al, 1994; Giaccone et al, 1996) including small groups of patients with local recurrence or distant metastases suggest that cisplatin containing regimens are active with an overall response rate ranging from 50% to 80%.

These promising data suggest testing chemotherapy in a neoadjuvant setting in order to improve the resection rate of patients with locally advanced disease. Six reports of primary chemotherapy followed by surgery in a total of 61 patients have been reviewed by Tomiak and Evans (1993). Forty-nine patients received a cisplatin-based regimen. The overall response to chemotherapy was 89%, with 19 complete remissions and 35 partial remissions. Twenty-two patients underwent surgery, and in 11 patients a complete resection was achieved – all 11 had been treated with a cisplatin-based regimen. Following the interesting results obtained in the largest series ever published from a single institution with the association of cisplatin, doxorubicin,

*Received 23 October 1998*
*Revised 21 April 1999*
*Accepted 30 April 1999*

*Correspondence to:* L Dogliotti vincristine and cyclophosphamide (ADOC), which reported a response rate of 92% in 37 advanced/metastatic patients (Fornasiero et al, 1991), we evaluated the ADOC scheme as preoperative chemotherapy in patients with stage III and IVa thymoma.

### PATIENTS AND METHODS

### Selection criteria

Patients presenting in our institution with locally advanced non-metastatic thymomas judged not amenable to surgical resection with radical intent, were considered in the present analysis. Eligibility criteria included: histological proof of thymoma, age less than 75 years, no history of malignancy other than basal cell carcinoma of the skin or in situ cervical cancer; measurable disease, performance status of 0–2 according to the ECOG (Eastern Cooperative Oncology Group) scale, adequate bone marrow reserve (leucocyte count > 3500 $\mu L^{-1}$, platelet count >100 000/$\mu L^{-1}$) and liver (bilirubin <1.5 mg $dl^{-1}$) and renal (serum creatinine <1.5 mg $dl^{-1}$, creatinine clearance >65 ml $min^{-1}$) function. Patients with local recurrent disease after radical surgery were also included. Exclusion criteria were the following: active angina pectoris, congestive heart failure, previous myocardial infarction, impaired ventricular ejection fraction, previous chemotherapy and/or radiation therapy, metastatic disease.

The pretreatment staging procedures included history and physical examination, chest X-ray, screening chemistries, electrocardiogram (ECG), computed tomography (CT) of the chest and abdomen, and anterior mediastinotomy with mediastinal resection biopsy. Other radiological investigations were performed, if necessary, to best estimate tumour extension.

Thymomas were classified according to the predominant cell type (Lewis et al, 1987): (a) lymphocytic, (b) epithelial, (c) mixed lympho-epithelial, (d) spindle cell. Stage was assessed according to Masaoka classification (Masaoka et al, 1981) as follows: I, macroscopically completely encapsulated; II, microscopic invasion into capsule and/or macroscopic invasion into surrounding fatty tissue, mediastinal pleura, and both; III, macroscopic invasion into contiguous viscera structure; IVa, pleural or pericardial dissemination; IVb, lymphogenous or hematogenous metastases.

### Treatment administration and response evaluation

Treatment consisted of intravenous administration of adriamycin (40 mg $m^{-2}$) and cisplatin (50 mg $m^{-2}$) given on day 1, vincristine (0.6 mg $m^{-2}$) on day 2 and cyclophosphamide (700 mg $m^{-2}$) on day 4 (ADOC). This scheme was repeated every 3 weeks. A chest radiography was planned after two chemotherapy courses, while a complete re-staging including CT scan of the chest and upper abdomen was performed after four cycles.

Response was assessed in accordance with the World Health Organization (WHO) criteria (Miller et al, 1981). Complete remission (CR) was defined as complete disappearance of all clinical, radiological and biochemical evidence of disease for a minimum of 1 month. Partial response (PR) was defined as a 50% or greater reduction in the sum of the product of the largest diameter and its perpendicular of all measurable lesions that lasted at least 4 weeks. Progressive disease (PD) was defined as an increase of at least 25% in the size of measurable lesions or the development of new lesions. Toxicity was graded according to the WHO criteria (Miller et al, 1981).

**Table 1** Patient characteristics

| | |
|---|---|
| No. | 16 |
| Males | 9 (56.2%) |
| Females | 7 (43.7%) |
| Age median (range) | 55 (28–69) |
| ECOG performance status | |
| 0 | 5 (31.2%) |
| 1 | 10 (62.5%) |
| 2 | 1 (6.3%) |
| Histology | |
| Lympho-epithelial | 12 (75.0%) |
| Epithelial | 2 (12.5%) |
| Spindle cell | 2 (12.5%) |
| Stage | |
| III | 10 (62.5%) |
| IVa | 6 (37.5%) |
| Previous surgery | 3 (18.7%) |

All patients with clinical CR or PR were addressed to surgery. Surgery was planned to be performed 3 or 4 weeks after the 4th chemotherapy cycle. In case of disease response but with the residual mass not amenable to surgery with radical intent, further chemotherapy was administered up to a maximum of six cycles. Resection was defined as radical if all macroscopic disease was removed and all surgical margins were free of tumour. Patients with malignant cells at post-operative histology received radiotherapy (45 Gy in fractionated doses) followed by two further ADOC cycles. Radiotherapy was planned instead of surgery in case of no response or in the presence of inoperable residual disease after chemotherapy.

Time to progression was measured from the start of chemotherapy until progression. Survival duration was measured from the date of treatment start until the day of death or last follow-up visit. Survival curves were plotted by the Kaplan–Meier technique and comparison was performed using the log-rank test. Statistical analysis was performed on an IBM-compatible personal computer using the SPSS-PC software (Nie et al, 1988).

### RESULTS

From October 1990 to November 1997, 16 consecutive patients (nine males and seven females) with stages III–IVa invasive thymoma entered the study. The clinical characteristics are listed in Table 1. Thirteen patients were evaluated at first diagnosis, while three were at first local relapse of disease 30, 42 and 85 months after radical surgery. No patients showed myasthenia gravis, lupus erythematosus, pure red aplasia or hypogammaglobulinaemia. One patient showed amenorrhea in association with steatorrhea, lasting one year prior to the thymoma detection.

Sixty-eight ADOC cycles were delivered (median 4, range 2–6). Toxicity was acceptable, nausea/vomiting, leukopenia and anaemia were the most frequent toxic events encountered, but were moderate in almost all patients. Only two patients displayed grade III leukopenia and one patient grade III nausea/vomiting. Anti-emetic treatment was metoclopramide in five patients and 5HT3 antagonists in the remaining 11. None of the patients on study received colony-stimulating factors (G-CSF or GM-CSF) or epoetin.

© 1999 Cancer Research Campaign

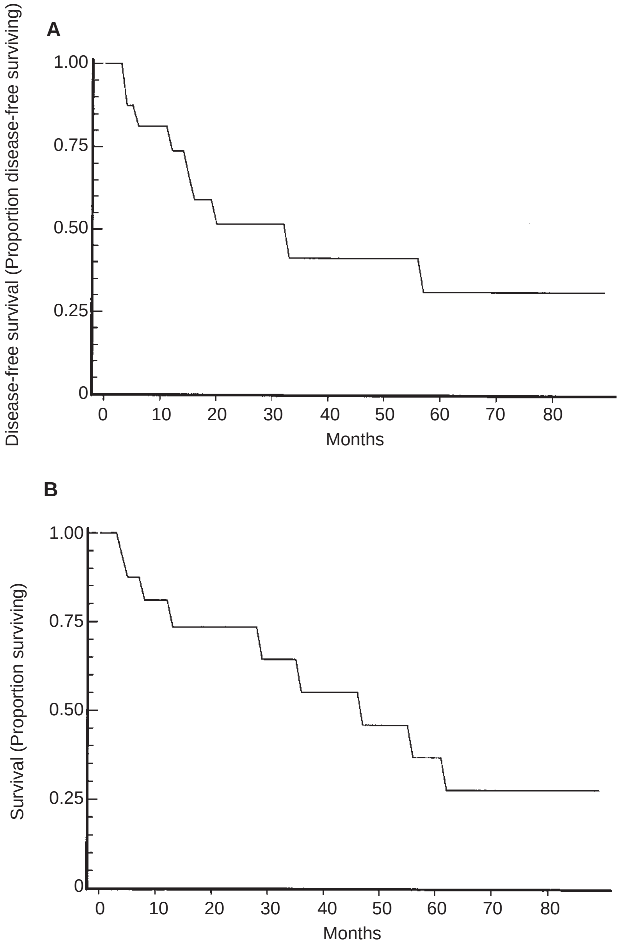

**Figure 1** Disease-free survival (**A**) and overall survival (**B**) in patients with locally advanced thymomas, submitted to primary chemotherapy

Dose reduction of all four drugs was performed in two patients due to leukopenia. Cisplatin only was reduced in one patient developing renal toxicity. Treatment delays (1 week) was performed in eight patients due to haematological toxicity. None of the patients interrupted treatment early due to toxicity. Treatment outcome of each patient is listed on Table 2. One patient attained a CR and 12 patients attained a PR, for an overall response rate of 13/16 (81.2%). Two patients showed a SD and only one progressed. Two patients: no. 9 and no. 13, received only three and two ADOC cycles, due to patient refusal and progressive disease respectively. The patient (no. 15) who achieved a CR after four chemotherapy cycles refused surgery and was addressed to radiotherapy. A disease recurrence occurred 2 months later leading to the patient's death.

Of 12 cases attaining a PR after four chemotherapy cycles, nine underwent radical surgery (five out of ten with stage III, and four out of six with stage IVa) and became disease free. Residual disease was observed in eight cases, while fibrosis was detected in one case. Two patients with PR and two with SD, at the end of the 4th cycle, were judged not operable with radical intent and received two further ADOC cycles, but afterwards they were still considered not amenable to surgery. Radiotherapy, either in adjuvant setting or instead of surgery, was performed in 13 patients, but was not performed in one case (no. 8) due to the absence of microscopic residual disease at post-chemotherapy surgery, and in the remaining two cases (nos 13 and 14) due to the rapid deterioration of the general condition as a consequence of progressive disease occurring after two and five ADOC cycles respectively.

The last date of follow-up was 15 September 1998, and at the time of writing nine patients have shown a disease progression and died of disease, while seven patients are still alive and disease free. Median disease-free interval (Figure 1A) and overall survival (Figure 1B) were 33.2 and 47.5 months respectively. Disease response to chemotherapy was associated with a relatively long survival prospect (median 57.2 months), whereas non-responding cases had a poor prognosis (median survival 12.5 months) ($P < 0.01$). Among the nine patients attaining the disease-free status after chemotherapy + surgery, progression occurred in four, while four cases survived more than 5 years (nos 4, 6, 8 and 12) (Table 2). The best outcome was observed in the patient obtaining a pathological complete response (fibrosis post-chemotherapy histology), who is still alive and disease-free after 8 years.

### DISCUSSION

Debulking surgery followed by radiotherapy is a well-recognized form of treatment in non-radically resectable invasive thymoma (Urgesi et al, 1992; Kohmann, 1997). Since thymoma is a chemosensitive neoplasm (Fornasiero et al, 1991; Macchiarini et al, 1991; Loeherer et al, 1994; Giaccone et al, 1996) it is logical to evaluate chemotherapy as a potential adjunct to surgery and radiotherapy in this clinical setting.

ADOC is the combination regimen most frequently used in Italy (Fornasiero et al, 1991). As a preoperative approach, in the Padua experience (Rea et al, 1993), it was found to be able to obtain a 100% response rate in 16 cases, 11 of them attaining a radical operation.

In the present series, the ADOC regimen confirmed its great activity with a response rate of 81%. The first six cases have been already described in a preliminary report by our group (Berruti et al, 1993). Patients obtaining a disease response to chemotherapy showed a consistent survival advantage in comparison to no-responders. It is well known that this difference may be unrelated to treatment and cannot be used for inferences about treatment effectiveness (Anderson et al, 1983); at any rate, these data suggest that response is a useful marker to predict patients destined to have a good prognosis.

It is known that the long-term survival rate of patients with locally advanced thymoma is mainly dependent on the possibility to attain a disease free status. The survival of stage III patients radically resected is similar, in fact, to that of stage II patients (Maggi et al, 1991). The resection rate represents, therefore, a surrogate parameter of treatment efficacy for patients submitted to primary chemotherapy. The per cent of patients attaining a disease-free status in our series was close to that reported elsewhere with the same scheme (Rea et al, 1993).

As mentioned in the introduction, about 50–60% of stage III cases can be radically resected, but no patients with stage IVa can benefit from surgery (Maggi et al, 1991). In the present experience, five out of ten cases with stage III, and four out of six with stage IVa, judged inoperable before chemotherapy, were radically resected. It is noteworthy that four ADOC cycles are sufficient to obtain the maximum results in terms of resection rate, as no patients judged unresectable after four cycles became resectable

*© 1999 Cancer Research Campaign*

**Table 2** Major patient characteristics and treatment outcome

| No. | Sex | Age | Histology | Stage | Previous therapy | No. of cycles | Response | Surgery | Post-chemotherapy histology | Radiotherapy | Time to progression (months) | Overall survival (months) |
|---|---|---|---|---|---|---|---|---|---|---|---|---|
| 1 | F | 62 | LE | III | – | 6 | PR | No | – | YES | 8+ | 8+ |
| 2 | F | 29 | E | IVa | Surgery | 4 | PR | Yes | Malignant cells | YES | 20 | 36 |
| 3 | F | 67 | LE | III | – | 4 | PR | Yes | Malignant cells | YES | 34 | 47 |
| 4 | M | 62 | LE | III | – | 4 | PR | Yes | Malignant cells | YES | 57 | 62 |
| 5 | M | 64 | LE | IVa | – | 6 | SD | No | – | YES | 15 | 29 |
| 6 | F | 36 | LE | III | – | 4 | PR | Yes | Malignant cells | YES | 69+ | 69+ |
| 7 | F | 47 | LE | III | – | 4 | PR | Yes | Malignant cells | YES | 16 | 56 |
| 8 | M | 28 | LE | IVa | – | 4 | PR | Yes | Fibrosis only | NO | 96+ | 96+ |
| 9 | M | 63 | SC | III | – | 3 | SD | No | – | YES | 12 | 14 |
| 10 | F | 36 | LE | III | Surgery | 4 | PR | Yes | Malignant cells | YES | 26+ | 26+ |
| 11 | M | 66 | SC | IVa | Surgery | 4 | PR | Yes | Malignant cells | YES | 22+ | 22+ |
| 12 | M | 44 | LE | IVa | – | 4 | PR | Yes | Malignant cells | YES | 65+ | 65+ |
| 13 | M | 64 | E | III | – | 2 | PD | No | – | NO | – | 5 |
| 14 | F | 38 | LE | III | – | 5 | PR | No | – | NO | 6 | 6 |
| 15 | M | 46 | LE | III | – | 4 | CR | No | – | YES | 6 | 8 |
| 16 | M | 69 | LE | IVa | – | 6 | PR | No | – | YES | 9+ | 9+ |

LE: Lympho-epithelial, E: epithelial, SC: spindle cells; PR: partial response, SD: stable disease, PD: progressive disease.

after two further courses. These data suggest that primary chemotherapy can improve the resection rate of locally advanced thymomas, but a larger number of patients and randomized trials are needed to this suggestion. Unfortunately, due to the rarity of the disease, such trials are very difficult to perform. In our hands, the ADOC combination regimen led to a low CR rate: 2/16 (12.5%), one clinical and one pathological. The patient attaining a clinical CR refused surgery and showed a disease progression after a few months, whereas the patient with pathological CR is still alive and disease-free more than 8 years after the end of chemotherapy. This case suggests that thymoma is potentially curable with chemotherapy alone, even though cure is obtained in the large minority of patients. It should be noted that ADOC is an old scheme, introduced more than 10 years ago (Fornasiero et al, 1991). It could be updated by increasing the dose of some single agents, particularly cisplatin, which is administered at lower doses than usually scheduled in modern combination regimens. In addition, as previously suggested, the efficacy of chemotherapy can be improved by the association of steroids (Fornasiero et al, 1991; Tomiak and Evans, 1993). A single institution experience, very recently published (Shin et al, 1998), showed a high response rate (92%) and a high resection rate (69%) in 13 patients with locally advanced thymoma submitted to a combination regimen consisting in cisplatin (90 mg $m^{-2}$), doxorubicin and cyclophosphamide plus oral prednisone. These results seemed to be better than those of the present study and this may be attributable to the higher cisplatin dose administered and the introduction of steroids in association with chemotherapy.

In a trial recently published, Loehrer et al (1997) reported a 69% response rate in 23 patients with limited-stage unresectable thymoma using a combination scheme including cyclophosphamide, doxorubicin and cisplatin (PAC). All patients were submitted to radiotherapy and none of them was submitted to surgery. Either the time to treatment failure or the overall survival were interesting. These results may suggest a combined chemo-radiotherapy without surgery in patients with unresectable disease ‘ab initio’. It should be remembered that thymomas have a great propensity for local relapse and on this basis we think that surgical resection, after significant response to chemotherapy, should be strongly advocated before radiotherapy in order to obtain the best local control.

To conclude, the present experience confirms that the ADOC scheme is active and manageable in the treatment of locally advanced thymoma. The results of the present study are similar to those obtained by another institution using the same scheme (Rea et al, 1993). An update of this regimen, with increase of cisplatin dose and the association of corticosteroids, can improve its efficacy. The impact of preoperative chemotherapy on overall survival cannot be assessed, due to the limited experiences up to now published and to the uncertain natural history of many patients with this disease. The elevated response rate obtained suggests that this approach should be offered not only to patients with locally advanced disease not amenable to surgery but also to those surgically resectable with a great deal of morbidity.

### REFERENCES

Anderson JR, Cain KC and Gelber RD (1983) Analysis of survival by tumor response. *J Clin Oncol*. **1**: 710–719

Berruti A, Borasio P, Roncari A, Gorzegno G, Mossetti C and Dogliotti L (1993) Neoadjuvant chemotherapy with adriamycin, cisplatin, vincristine and cyclophosphamide (ADOC) in invasive thymomas: results in six patients. *Ann Oncol* **4**: 429–431

Curran WJ, Kornstein MJ, Brooks JJ and Turrisi T (1988) Invasive thymoma: the role of mediastinal irradiation following complete or incomplete surgical resection. *J Clin Oncol* **6**: 1722–1727

Decarli A, La Vecchia C, Cislaghi C and Negri E (1998) Cancer mortality in Italy, 1994, and an overview of trends from 1955 to 1994. *Tumori* **84**: 312–334

Fornasiero A, Daniele O, Ghiotto C, Piazza M, Fiore-Donati L, Calabro’ F, Rea F and Fiorentino MV (1991) Chemotherapy for invasive thymoma. A 13-year experience. *Cancer* **68**: 30–33

Giaccone G, Musella R, Bertetto O, Donadio M and Calciati A (1985) Cisplatin-containing chemotherapy in the treatment of invasive thymoma: report of five cases. *Cancer Treat Rep* **69**: 695–697

Giaccone G, Ardizzoni A, Kirkpatrick A, Clerico M, Sahmoud T and Van Zandwijk N (1996) Cisplatin and etoposide combination for locally advanced or metastatic thymoma: a phase II study of the European Organisation for Research and Treatment of Cancer Lung Cancer Cooperative Group. *J Clin Oncol* **14**: 814–820

*© 1999 Cancer Research Campaign*

Kirschner PA (1990) Reoperation for thymoma: report of 23 cases. *Ann Thorac Surg* **49**: 550–555

Kohman JL (1997) Controversies in the management of malignant thymoma. *Chest* **112**: 296S–300S

Lewis JE, Wick MR, Scheithauer BW, Bernatz P and Taylor WF (1987) Thymoma: a clinicopathologic review. *Cancer* **60**: 2727–2743

Loehrer P, Kim KM, Aisner SC, Livingston R, Einnhorn LH, Johnson D and Blum R (1994) Cisplatin plus doxorubicin plus cyclophosphamide in metastatic recurrent thymoma: final results of an intergroup trial. *J Clin Oncol* **12**: 1164–1168

Loehrer P, Chen M, Kim Km, Aisner SC, Einnhorn LH, Livingston R and Johnson D (1997) Cisplatin, doxorubicin, and cyclophosphamide plus thoracic radiation therapy for limited-stage unresectable thymoma: an intergroup trial. *J Clin Oncol* **15**: 3093–3099

Macchiarini P, Chella A, Ducci F, Rossi B, Testi C, Bevilacqua G and Angeletti A (1991) Neoadjuvant chemotherapy, surgery, and postoperative radiation therapy for invasive thymoma. *Cancer* **68**: 706–713

Maggi G, Giaccone G, Donadio M, Ciuffreda L, Dalesio O, Leria G, Trifiletti G, Casadio C, Palestro G and Mancuso M (1986) Thymomas: a review of 169 cases with particular reference to results of surgical treatment. *Cancer* **58**: 765–776

Maggi G, Casadio C, Cavallo A, Cianci R, Molinatti M and Ruffini E (1991) Thymoma: results of 241 operated cases. *Ann Thorac Surg* **51**: 152–156

Masaoka A, Monden Y, Nakahara K and Tanioka T (1981) Follow-up study of thymoma with reference to their clinical stages. *Cancer* **4**: 2485–2492

Miller AB, Hoogstraten B, Staquet M and Winkler A (1981) Reporting results of cancer treatment. *Cancer* **47**: 207–214

Nie NH, Hull CH and Jeakins JG (1988) *Statistical Package for the Social Sciences*. SPSS Inc, Chicago IL

Pescarmona E, Rendina EA, Venuta F, D'Arcangelo E, Pagani M, Ricci C, Ruco LP and Baroni CD (1990) Analysis of prognostic factors and clinicopathological staging of thymoma. *Ann Thorac Surg* **50**: 534–538

Pollack A, El-Naggar AK, Cox JD, RO JY, Sahin A and Komaki R (1991) Thymoma: the prognostic significance of flow cytometric DNA analysis. *Cancer* **69**: 1702–1709

Rea F, Sartori F, Loy M, Calabro' F, Fornasiero A, Daniele O and Altavilla G (1993) Chemotherapy and operation for invasive thymoma. *J Thorac Cardiovasc Surg* **106**: 543–549

Schmidt R, Monig P, Selzner M and Krug B (1997) Surgical therapy of malignant thymoma. *J Cardiovasc Surg* **38**: 317–322

Shin DM, Walsh GL, Komaki R, Putnam JB, Nesbitt J, Ro JY, Shin HJC, Ki KH, Wimberly A, Pisters KMW, Schrump D, Gregurich MA, Cox JD, Roth JA and Hong WK (1998) A multidisciplinary approach to therapy for unresectable malignant thymoma. *Ann Intern Med* **129**: 100–104

Tomiak EM and Evans WK (1993) The role of chemotherapy in invasive thymoma: a review of the literature and considerations for future clinical trials. *Crit Rev Oncol/Hematol* **15**: 113–242

Urgesi A, Monetti U, Rossi G, Ricardi U, Maggi G and Sannazzari GL (1992) Aggressive treatment of intrathoracic recurrences of thymoma. *Radiother Oncol* **24**: 221–225

Verdecchia A and the Itacare working group (1997) Survival in adult Italian cancer patients. *Tumori* **83**: 39–42

Verley JM and Hollmann KH (1985) Thymoma: a comparative study of clinical stages, histologic features, and survival in 200 cases. *Cancer* **55**: 1074–1086

Yagi K, Hirata T, Fukuse T, Hiroyasu Y, Kenji I, Osamu I, Hiroshi M, Minoru A, Shigeki H and Hiromi W (1996) Surgical treatment for invasive thymoma especially when the superior vena cava is invaded. *Ann Thorac Surg* **61**: 521–524

*© 1999 Cancer Research Campaign*
